# Supplementary material for: LARP7 family proteins have conserved function in telomerase assembly
Source: Nat Commun. 2018 Feb 8;9:557. doi: 10.1038/s41467-017-02296-4 (PMC5805788; doi:10.1038/s41467-017-02296-4)
Supplement: Supplementary file 2 — Supplementary Information [file 41467_2017_2296_MOESM2_ESM.pdf]

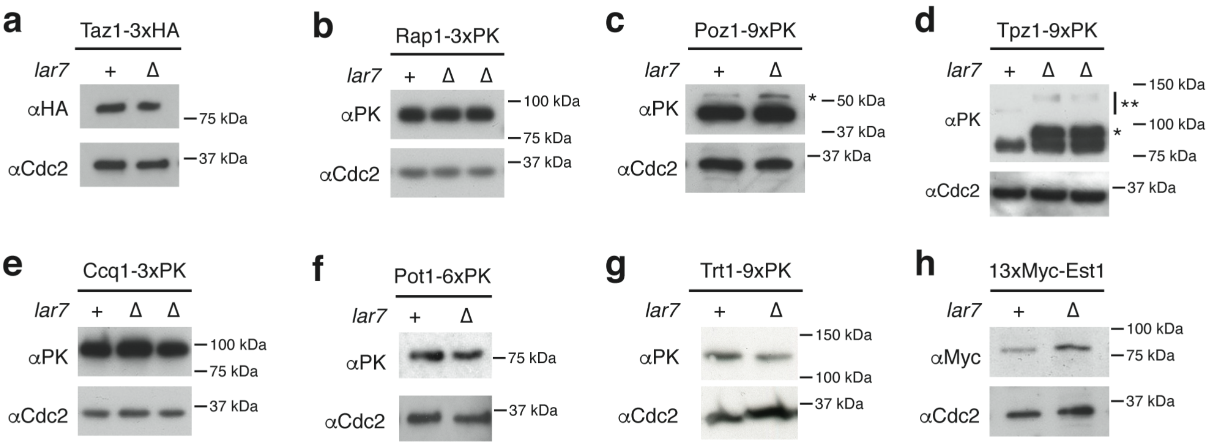

Supplementary Figure 1 – Expression of the telomeric proteins and telomerase subunits in *lar7*Δ

Strains carrying indicated tagged proteins Taz1-3xHA (a), Rap1-3xPK (b), Poz1-9xPK (c), Tpz1-9xPK (d), Ccq1-3xPK (e), Pot1-6xPK (f), Trt1-9xPK (g) and 13xMyc-Est1 (h), were extracted using a 20% TCA extraction method and the extract was subjected to Western blot. Cdc2 was used as a loading control. Stability of examined proteins were not affected in the absence of Lar7. Poz1-9xPK (c) exhibited a slow migrating form (*), which was more visible in *lar7*Δ. Tpz1 (d) also exhibited a slow migrating form (*) in *lar7*Δ. In the absence of Lar7, telomeres are shortened, which presumably causes phospho-modification. Tpz1 is also SUMO-modified, which appeared as a faint band (**) in both wt and *lar7*Δ cells [1].

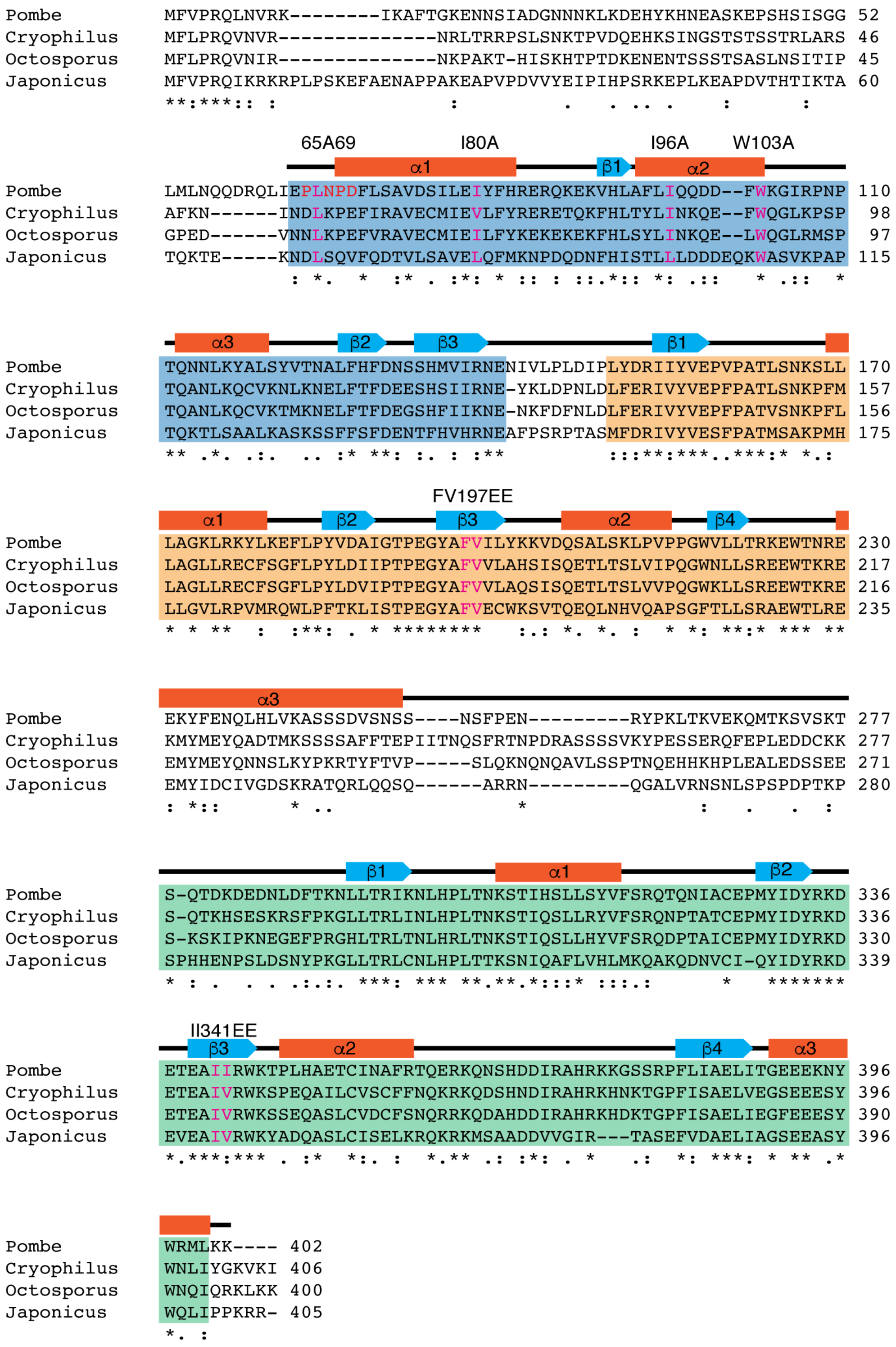

Supplementary Figure 2 – Clustal Omega sequence alignment of Lar7 from four fission yeast species

Lar7 protein of *S. pombe*, *S. Cryophilius*, S. *Octosporus* and *S. Japonicus* were aligned using Clustal Omega [2]. Asterisks, colons and the full stops below the alignment indicate conserved residues, residues with strongly similar properties and residues with weaker similar properties, respectively. The conserved La-motif, RMM1 and RMM2 domains are highlighted in blue, orange and green boxes respectively. The orange and cyan boxes above the alignment indicate predicted secondary structure of helices and sheets. Mutations generated in this study are indicated above and the targeted conserved residues are highlighted with magenta.

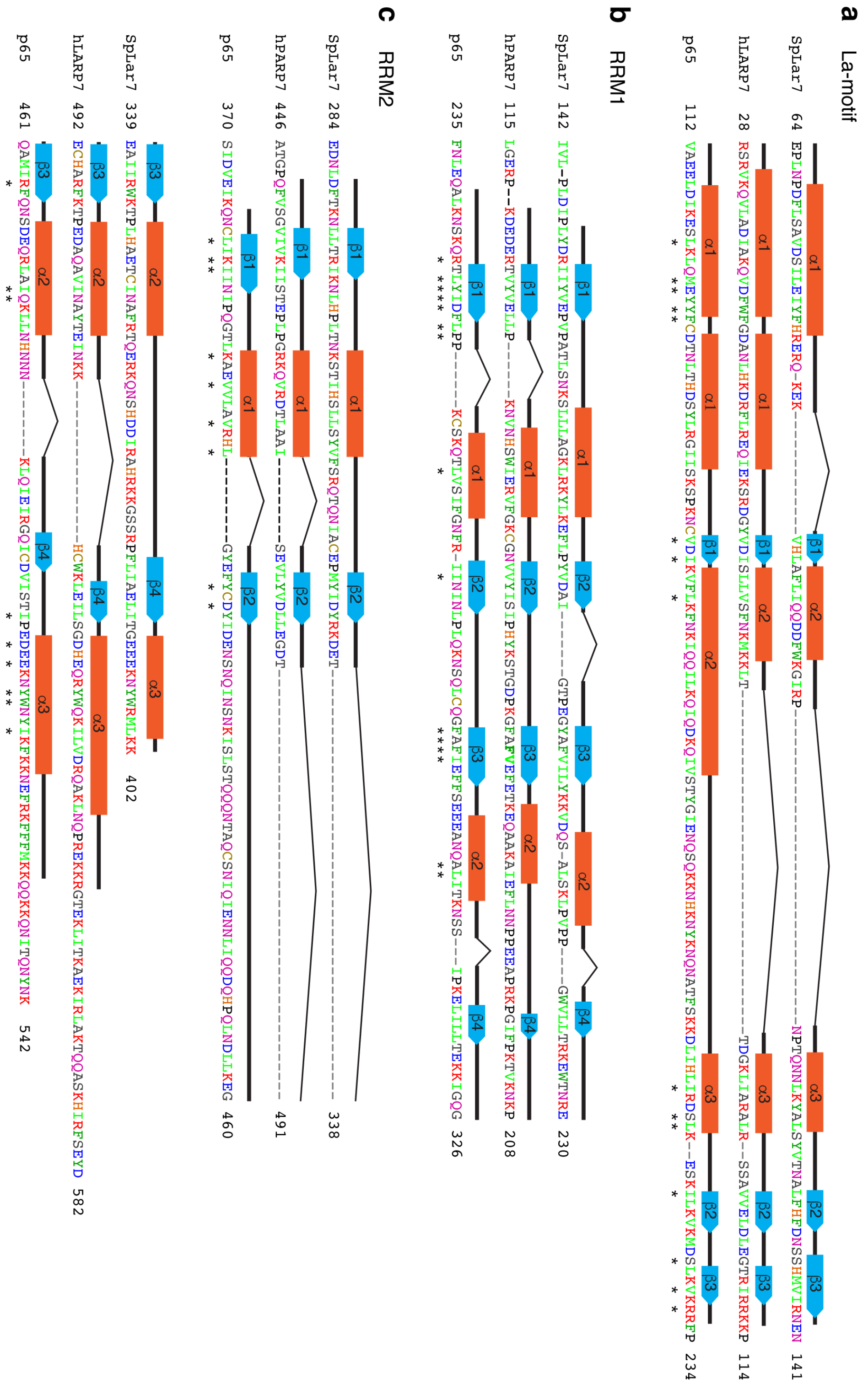

Supplementary Figure 3 – HHpred-based alignment of the La-motif, RMM1 and RMM2 domains between *S. pombe* Lar7, human LARP7 and *Tetrahymena* p65

Lar7, hLARP7 and p65 were analysed using HHpred and the La-motif (a), RMM1 (b) and RMM2 (c) were aligned based on the crystal structures of the La-module (La-motif and RMM1) from hLARP7 (1-208aa, NDB: 4wkr [3]) and RMM2 from hLARP7 (445-561 aa, NDB: 5knw [4]) as references. The asterisks below the alignment indicate conserved residues or the residues with strongly similar properties. The orange and cyan boxes above the alignment indicate predicted secondary helix and sheet structures.

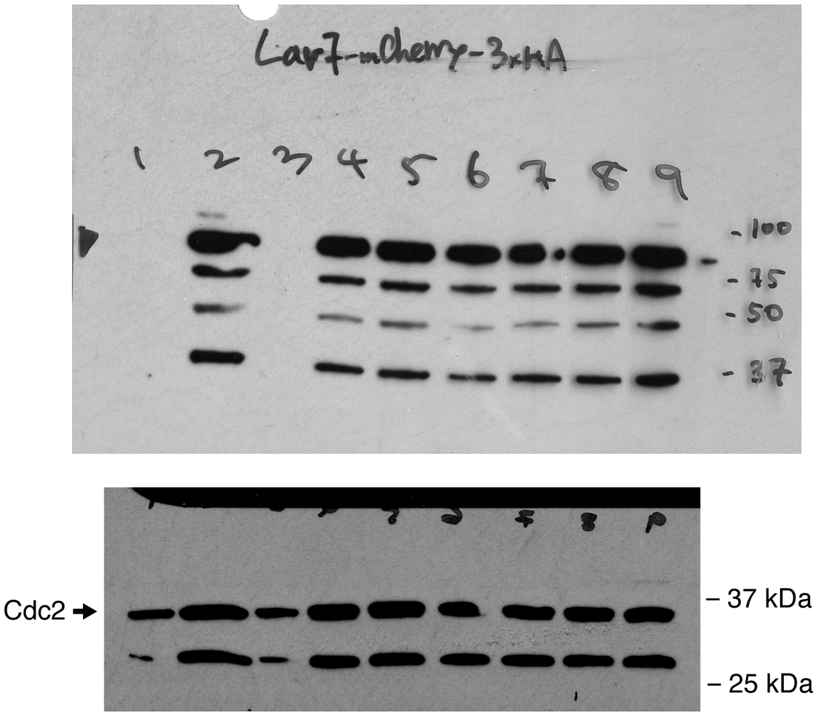

Supplementary Figure 4 – Immunoblot of the lysate from cells carrying HA-tagged Pof8

Original blot image of immunoblot with anti-HA for Pof8 mutant proteins. Top bands above 75kDa are expected molecular weight of the intact Pof8-mCherry-3xHA fusion protein (arrowhead), and are shown in Figure 2b. Three faster migrating bands are expected to be degraded forms of 3xHA fused proteins as they are not detected in wild type and lar7Δ strains. Lane 1, no-tag wild type; lane 2, HA-tagged Pof8; Lane 3, *pof8Δ*; Lanes 4-9, HA-tagged mutant Pof8 (From left: *65A69*, *I80A*, *I97A*, *W103A*, *FV197EE* and *II341EE*)

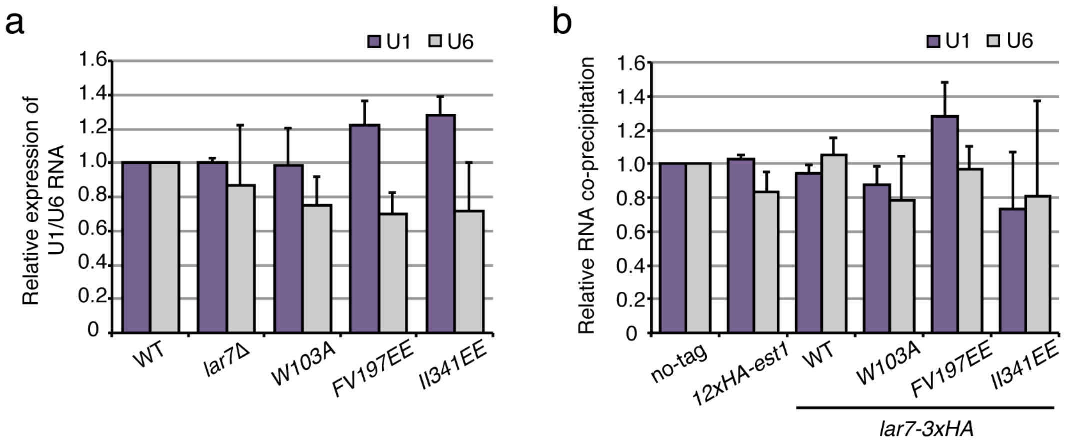

Supplementary Figure 5 – Lar7 does not interact with U1 or U6 non-coding RNAs
(a) U1 and U6 mRNA was quantified in *lar7Δ* or mutant cells using RT-qPCR and normalised first to actin and then to the expression in wild-type cells. Data represented as a mean of 2 independent experiments. Error bars show standard deviation. (b) The presence of U1 and U6 mRNA in lysates following immunoprecipitation using α-HA was measured using reverse-transcriptase quantitative polymerase chain reaction (RT-qPCR) and normalised first to actin mRNA and shown as relative enrichment relative to wild-type untagged Lar7. Data is represented as a mean of two independent experiments. Error bars show standard deviation.

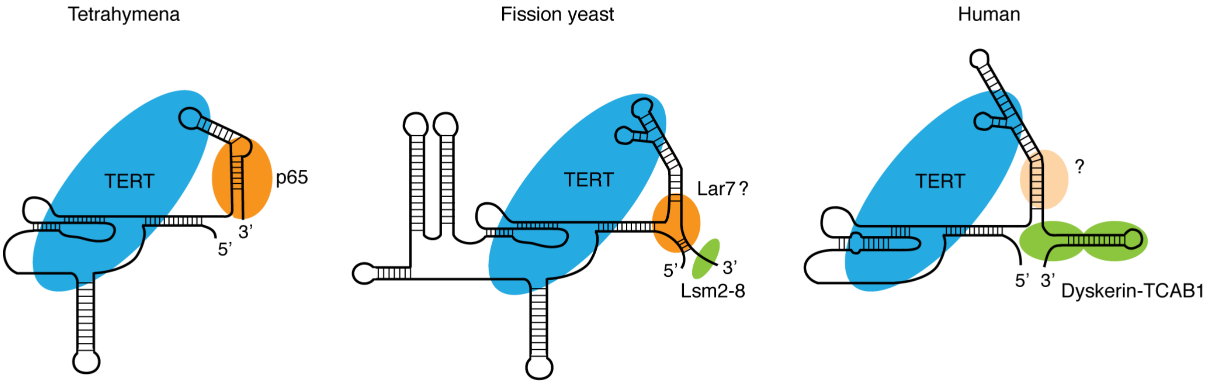

Supplementary Figure 6 – Proposed role of fission yeast Lar7 and potential conserved mechanism in telomerase assembly

(Left) *Tethrahymena* p65 binds to the stem of the STE domain at the 3’ end to facilitate interaction of TERT with both the pseudoknot and STE domains. (Middle) A similar role is expected in fission yeast Lar7. Whereas the 3’ end of TER1 is protected by the Lsm proteins, Lar7 may bind to the stem of the STE domain to support interaction of Trt1 with both the pseudoknot and STE domains. (Right) Similarities between telomerase RNAs in fission yeast and humans implicate LARP7 (or a similar) protein in a similar role.

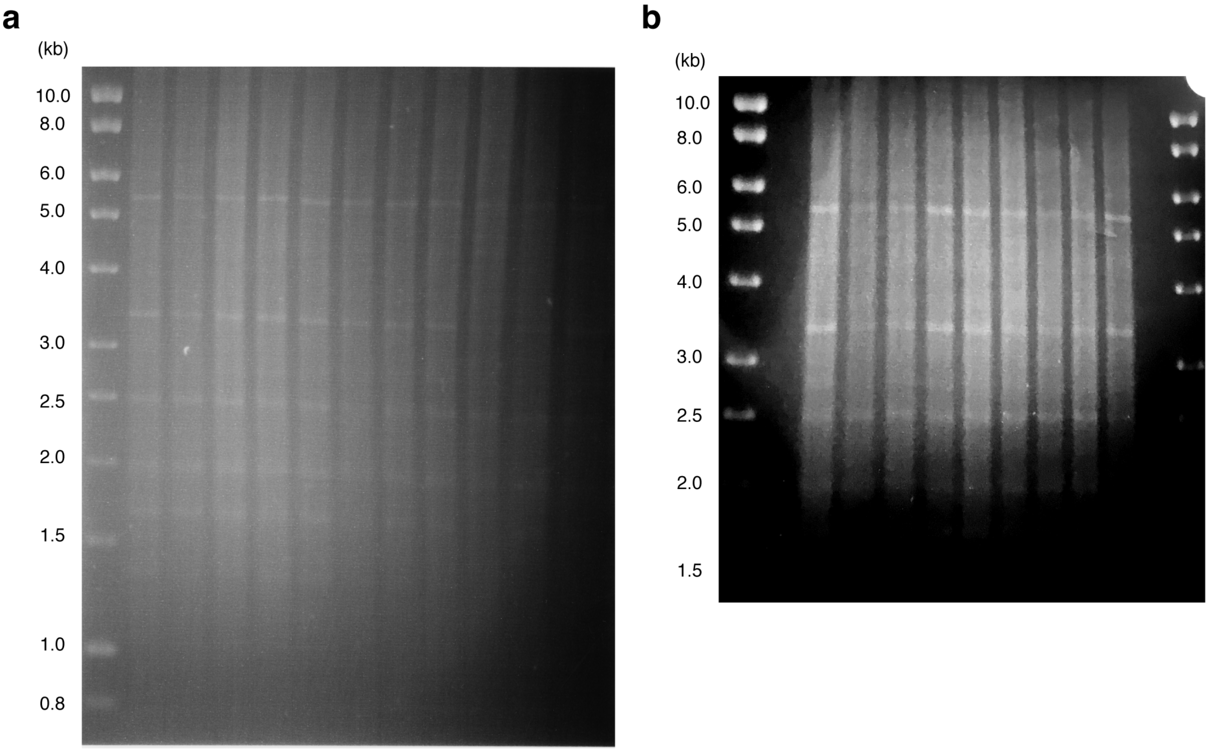

Supplementary Figure 7 – uncropped images of DNA staining loading control in (a) Fig. 1b and (b) Fig. 4b.
DNA bands around 5-6 kb are cropped and shown.

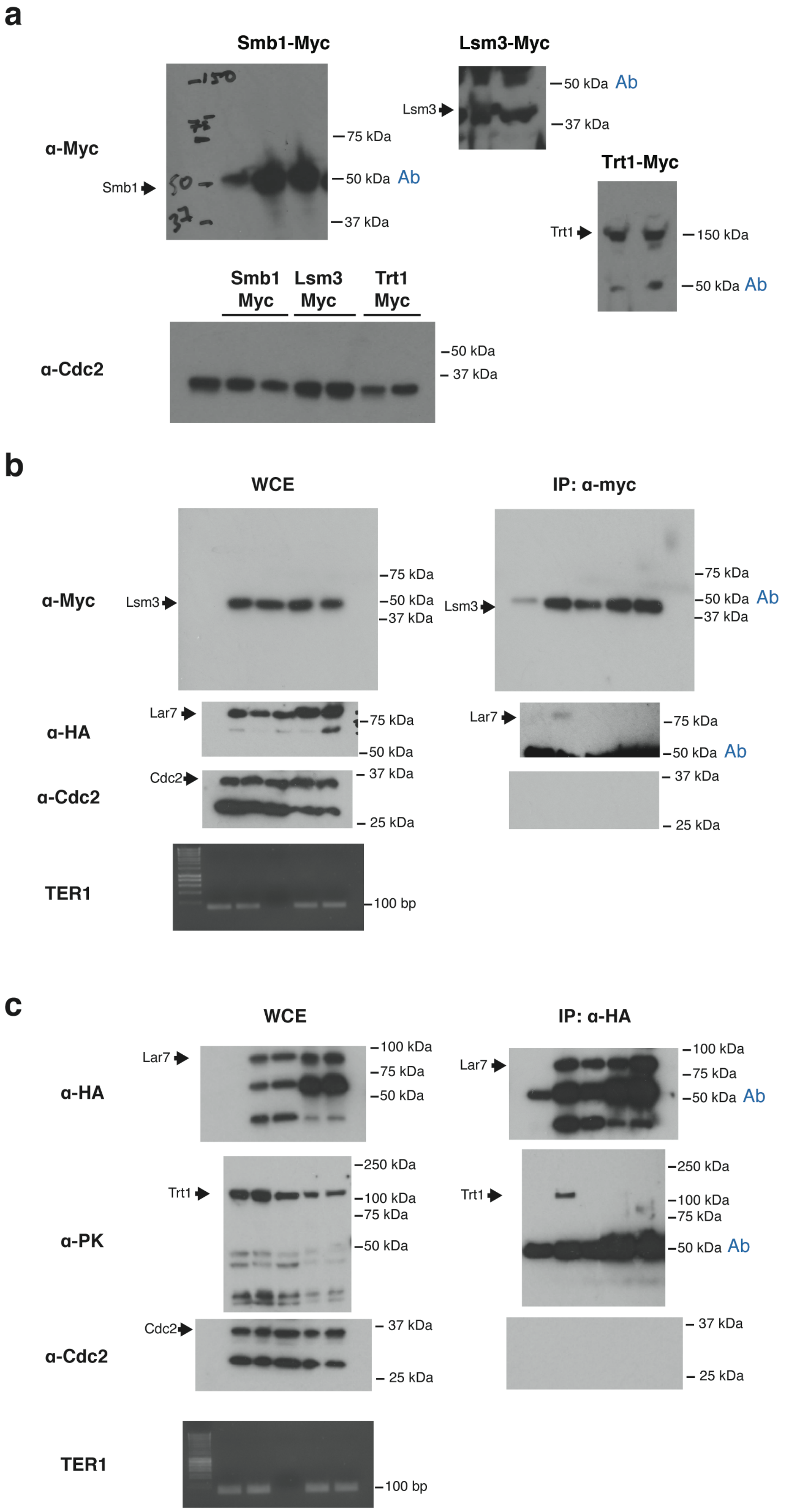

Supplementary Figure 8 – wider area of blot images used in Figure 5.
Predicted size of C-terminally tagged proteins, Smb1-13xMyc, Lsm3-13xMyc, Trt1-13xMyc, Trt1-9xPK and Lar7-mCherry-3xHA are indicated. For immunoprecipitated samples, antibody band (Ab) can be detected at around 50 kDa. Intact protein bands are shown in Figure 5.

Supplementary Table 1 – Fission yeast strain list used for the study

Strain no.	Genotype	Figure
1	h^-	WT control
3	h^- leu1-32 ura4-D18	WT control
1626	h^-/h^+ ade6-M210/M216 lar7::natMX6/+	1a
1654	h^- ade6-M210 lar7::natMX6	1, 3, 4, S4, S5a
2465	$h^?$ ade6-M? trt1::hygMX6	1b, 3a
1693	h^- ade6-M216 lar7::kanMX6 trt1::hygMX6	1b
707	h^+ ade6-M216 leu1-32 ura4-D18 rad51::kanMX6	1b
1749	h^- ade6-M216 lar7::natMX6 rad51::kanMX6	1b
209	h^- ade6-M210 leu1-32 ura4-D18 his3-D1 taz1::hygMX6	1c
1641	h^+ lar7::kanMX6 taz1::hygMX6	1c
596	h^- rap1::kanMX6	1c
1696	h^+ ade6-M216 lar7::natMX6 rap1::kanMX6	1c
591	h^- rif1::kanMX6	1c
1694	h^- ade6-M216 lar7::natMX6 rif1::kanMX6	1c
3894	h^+ leu1-32 ura4-D18 lar7-mCherry-3xHA:natCX	2, 3c, S4, S5
3898	h^+ leu1-32 ura4-D18 lar7(65AAAAA69)-mCherry-3xHA:natCX	2, S4
3899	h^+ leu1-32 ura4-D18 lar7(I80A)-mCherry-3xHA:natCX	2, S4
3900	h^+ leu1-32 ura4-D18 lar7(I97A)-mCherry-3xHA:natCX	2, S4
3901	h^+ leu1-32 ura4-D18 lar7(W103A)-mCherry-3xHA:natCX	2, 3b, 3c, S4, S5
3902	h^+ leu1-32 ura4-D18 lar7(FV197EE)-mCherry-3xHA:natCX	2, 3b, 3c, S4, S5
3903	h^+ leu1-32 ura4-D18 lar7(II341EE)-mCherry-3xHA:natCX	2, 3b, 3c, S4, S5
297	h^{-smt0} leu1-32 ura4-D18 est1::hygMX6:P^{est1}>24xHA-est1	3c, S4b
3715	h^- ter1::TKnatAX	3a
3835	h^+ leu1-32 ura4-D18 lar7::kanMX6	4
3932	h^- ade6-M210 leu1-32 ura4-D18 his3-D1 rrp6::$ura4^+$	4
3950	h^- ade6-M210 leu1-32 ura4-D18 his3-D1 rrp6::$ura4^+$ lar7::kanMX6	4
3805	h^- ter1::P^{nmt41}>ter1:aur^R	4
3847	h^- lar7::kanMX6 ter1::P^{nmt41}>ter1:aur^R	4
4051	h^- leu1-32 ura4-D18 his3-D1 rrp6::$ura4^+$ ter1::P^{nmt41}>ter1:aur^R	4
4052	h^- leu1-32 ura4-D18 his3-D1 rrp6::$ura4^+$ lar7::kanMX6 ter1::P^{nmt41}>ter1:aur^R	4
3977	h^- leu1-32 ura4-D18 smb1-13xmyc:hygMX6	5a
3983	h^- leu1-32 ura4-D18 lar7::kanMX6 smb1-13xmyc:hygMX6	5a
3969	h^+ ura4-D18 lar7::kanMX6 lsm3-13xMyc:hygMX6	5a
3951	h- leu1-32 ura4-D18 lsm3-13xMyc:hygMX6	5a, 5b
4019	h+ leu1-32 ura4-D18 lsm3-13xMyc:hygMX6 lar7-mCherry-3xHA:natCX	5b
4020	h^- leu1-32 ura4-D18 lsm3-13xMyc:hygMX6 lar7(W103A)-mCherry-3xHA:natCX	5b
4021	h^- leu1-32 ura4-D18 lsm3-13xMyc:hygMX6 lar7(LV197EE)-mCherry-3xHA:natCX	5b
1629	h^- leu1-32 ura4-D18 his3-D1 trt1-9xPK:kanMX6	5c, S1g
3993	h^- leu1-32 ura4-D18 his3-D1 trt1-9xPK:kanMX6 lar7-mCherry-	5c

	3xHA:natCX	
3994	*h^- leu1-32 ura4-D18 his3-D1 trt1-9xPK:kanMX6 lar7(W103A)-mCherry-3xHA:natCX*	5c
3995	*h^- leu1-32 ura4-D18 his3-D1 trt1-9xPK:kanMX6 lar7(FV197EE)-mCherry-3xHA:natCX*	5c
1381	*h^+ pof8-13xMyc:kanMX6*	5d
1962	*h^+ leu1-32 ura4-D18 trt1-13xMyc:natMX6*	5d
2489	*h+ ade6-M216 leu1-32 ura4-D18 lar7::kanMX6 trt1-13xMyc:natMX6*	5d
740	*h^+ taz1-3xHA:kanMX6*	S1a
4072	*h^+ ade6-M210 lar7::natMX6 taz1-3xHA:kanMX6*	S1a
1427	*h^- ade6-M216 rap1-3xPK:natMX6*	S1a
1787	*h^- lar7::kanMX6 rap1-3xPK:natMX6*	S1a
455	*h^+ leu1-32 ura4-D18 poz1-9xPK:kanMX6*	S1c
1779	*h^+ ade6-M210 leu1-32 ura4-D18 lar7::natMX6 poz1-9xPK:kanMX6*	S1c
752	*h^- ade6-M210 leu1-32 ura4-D18 tpz1-9xPK:kanMX4*	S1d
1946	*h^+ ade6-M216 lar7::natMX6 tpz1-9xPK:kanMX4*	S1d
1406	*h^- ura4-D18 ccq1-3xPK:hygMX6*	S1e
1782	*h^+ ura4-D18 lar7::natMX6 ccq1-3xPK:hygMX6*	S1e
748	*h^+ ade6-M210 leu1-32 ura4-D18 pot1-6xPK:kanMX4*	S1f
1952	*h^- ade6-M216 ura4-D18 lar7::natMX6 pot1-6xPK:kanMX4*	S1f
1777	*h^+ ade6-M216 lar7::natMX6 trt1-9xPK:kanMX6*	S1g
1942	*h^+ lar7::natMX6 est1::hygMX6:P^{est1}>13xMyc-est1*	S1h
296	*h^{-smt0} leu1-32 ura4-D18 est1::hygMX6:Pest1>13xMyc-est1*	S1h

Supplementary references

1. Miyagawa K, et al. SUMOylation regulates telomere length by targeting the shelterin subunit Tpz1(Tpp1) to modulate shelterin-Stn1 interaction in fission yeast. *Proceedings of the National Academy of Sciences of the United States of America* **111**, 5950-5955 (2014).

2. Sievers F, et al. Fast, scalable generation of high-quality protein multiple sequence alignments using Clustal Omega. *Mol Syst Biol* **7**, 539 (2011).

3. Uchikawa E*, et al.* Structural insight into the mechanism of stabilization of the 7SK small nuclear RNA by LARP7. *Nucleic acids research* **43**, 3373-3388 (2015).

4. Eichhorn CD, Chug R, Feigon J. hLARP7 C-terminal domain contains an xRRM that binds the 3' hairpin of 7SK RNA. *Nucleic acids research* **44**, 9977-9989 (2016).
